# Radiomic Features Applied to Contrast Enhancement Spectral Mammography: Possibility to Predict Breast Cancer Molecular Subtypes in a Non-Invasive Manner

**DOI:** 10.3390/ijms232315322

**Published:** 2022-12-05

**Authors:** Luca Nicosia, Anna Carla Bozzini, Daniela Ballerini, Simone Palma, Filippo Pesapane, Sara Raimondi, Aurora Gaeta, Federica Bellerba, Daniela Origgi, Paolo De Marco, Giuseppe Castiglione Minischetti, Claudia Sangalli, Lorenza Meneghetti, Giuseppe Curigliano, Enrico Cassano

**Affiliations:** 1 Breast Imaging Division, Radiology Department, IEO European Institute of Oncology IRCCS, 20141 Milan, Italy; 2 Breast Radiology, Fondazione IRCCS Istituto Nazionale dei Tumori, 20133 Milano, Italy; 3 Department of Radiological and Hematological Sciences, Catholic University of the Sacred Heart, Largo Francesco Vito 1, 00168 Rome, Italy; 4 Molecular and Pharmaco-Epidemiology Unit, Department of Experimental Oncology, IEO IRCCS, 20139 Milan, Italy; 5 Medical Physics Unit, IEO European Institute of Oncology IRCCS, Via Ripamonti 435, 20141 Milan, Italy; 6 School of Medical Physics, University of Milan, Via Celoria 16, 20133 Milan, Italy; 7 Data Management, European Institute of Oncology IRCCS, 20141 Milan, Italy; 8 Department of Oncology and Hemato-Oncology, University of Milano, 20122 Milano, Italy; 9 Division of New Drugs and Early Drug Development for Innovative Therapies, European Institute of Oncology, IRCCS, 20141 Milan, Italy

**Keywords:** radiomics, CESM, molecular subtypes, breast cancer

## Abstract

We aimed to investigate the association between the radiomic features of contrast-enhanced spectral mammography (CESM) images and a specific receptor pattern of breast neoplasms. In this single-center retrospective study, we selected patients with neoplastic breast lesions who underwent CESM before a biopsy and surgical assessment between January 2013 and February 2022. Radiomic analysis was performed on regions of interest selected from recombined CESM images. The association between the features and each evaluated endpoint (ER, PR, Ki-67, HER2+, triple negative, G2–G3 expressions) was investigated through univariate logistic regression. Among the significant and highly correlated radiomic features, we selected only the one most associated with the endpoint. From a group of 321 patients, we enrolled 205 malignant breast lesions. The median age at the exam was 50 years (interquartile range (IQR) 45–58). NGLDM_Contrast was the only feature that was positively associated with both ER and PR expression (*p*-values = 0.01). NGLDM_Coarseness was negatively associated with Ki-67 expression (*p*-value = 0.02). Five features SHAPE Volume(mL), SHAPE_Volume(vx), GLRLM_RLNU, NGLDM_Busyness and GLZLM_GLNU were all positively and significantly associated with HER2+; however, all of them were highly correlated. Radiomic features of CESM images could be helpful to predict particular molecular subtypes before a biopsy.

## 1. Introduction

Breast cancer (BC) is the most commonly occurring cancer in women and represents the fifth leading cause of death in the global population, with an estimated 2.3 million cases in 2020. BC has recently become the most diagnosed cancer, surpassing lung cancer; at least one in eight women receive a breast cancer diagnosis in her lifetime [1,2]. Therefore, early detection and diagnosis of breast lesions have always been a challenge for breast radiologists due to the importance of improving the quality of life and increasing the survival rate of patients with BC. Full-field digital mammography (FFDM) is currently the main breast screening method; however, FFDM sensitivity is limited, in particular in women with dense breasts; according to some data reported in the literature, FFDM could miss BC in approximately 20% of positive cases [3].

Breast ultrasound (US) is particularly suitable for studying dense breasts, but its overall diagnostic efficacy is affected by interpretative issues: a restricted field of view and high operator dependency [4]. Furthermore, the difficulty of US (compared with mammography) when it comes to depicting microcalcifications reduces the sensitivity in those forms of breast malignancies that present only as microcalcifications [5].

A relatively new technique derived from FFDM named contrast-enhanced spectral mammography (CESM) has been developed to improve the detection and management of breast cancer diagnosis in dense breasts [6].

CESM takes advantage of the differential enhancement between neoplastic and normal tissue after the injection of an iodinated contrast agent. CESM is based on the acquisition of two pairs of images: low energy (LE) and high energy (HE). The high-energy image highlights contrast medium uptake well, while the low-energy image is similar to a conventional mammogram. The images are then recombined by subtracting the signal from the glandular tissue and better highlighting any contrast medium uptake [7].

The excellent performance of CESM in the diagnosis and management of breast malignancies was already demonstrated in many studies [8]. CESM, in particular, showed excellent sensitivity in the diagnosis of breast neoplasms comparable with that of MRI [9].

Moreover, CESM seems to be less affected by the problem of false positives, which appears to be one of the main issues of breast MRI [10]. While the role of CESM use in clinical practice appears increasingly established, far fewer studies investigated the clinical possibilities of CESM combined with radiomics, where radiomics is currently one of the main emerging disciplines in radiological studies due to its ability to translate images into high-dimensional data that can reflect not only macroscopic but also microscopic properties of tissues. Radiomics is a quantitative approach to medical imaging that analyses the grey values of a radiological image with the extraction, via a computer algorithm, of quantitative information that was not obtainable from conventional qualitative analysis; information extracted with radiomics in the form of features can be associated with certain clinical parameters, and thus, can influence medical choices regarding patients [11,12,13,14]. One of the most important clinical parameters of breast neoplasms is the receptor pattern. Different receptor arrangements are associated with changes in therapeutic management and prognosis. Our study aimed to investigate the association of radiomic features of CESM enhancement with a specific receptor pattern of breast neoplasms. The possibility of predicting a specific receptor pattern prior to biopsy could have important clinical repercussions: in a context, such as a breast pathology, that is characterized by a high number of patients, we could have the possibility to identify patients at higher risk of having more severe pathology to be referred first to biopsy assessment and the management of the breast lesion pathway. Patients in whom we expect more severe pathology could be referred, for example, to a preferential waiting list.

## 2. Results

**Figure 1:**
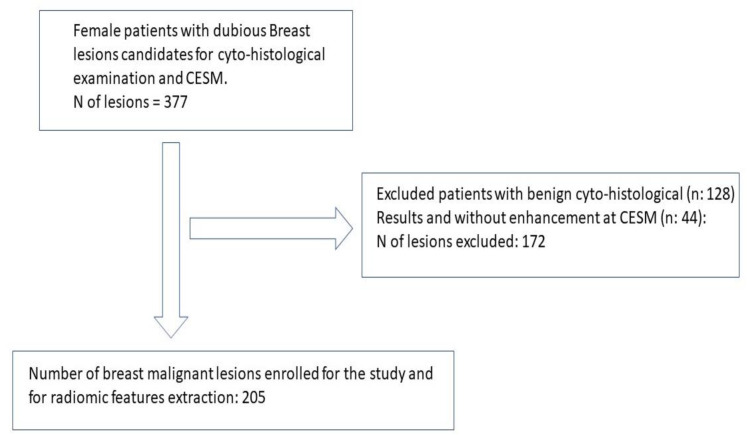
Lesion selection flow chart. Selection of breast pathological lesions for radiomic analyses.

From a group of 321 enrolled patients, we selected 205 malignant breast lesions. A flow chart of the criteria used to select the breast lesions for radiomic analysis is shown in Figure 1.

The descriptive characteristics of the patients and their lesions are summarized in Table 1.

The median age at the exam was 50 years (IQR 45–58). The median compression force was 70 N (interquartile range (IQR) 60–81). Most of the lesions were masses (84%), less frequently microcalcifications (8.3%). The most frequent BI-RADS assigned to the lesions by the radiologists was 4c (68%). Most of the lesions were identified in dense breasts (ACR C, 68%). The background parenchymal enhancement was minimal in the majority of cases (62%). The intensity of enhancement was shown to be marked in most cases (45%). The enhancement median size of the lesion was 17 mm (IQR: 11–30). In 21 (10.2%) cases, the lesions were in situ, whereas 89.7% of patients had an invasive lesion (the histological results of surgery are summarized in Supplementary Table S1).

### Features

**Figure 2:**
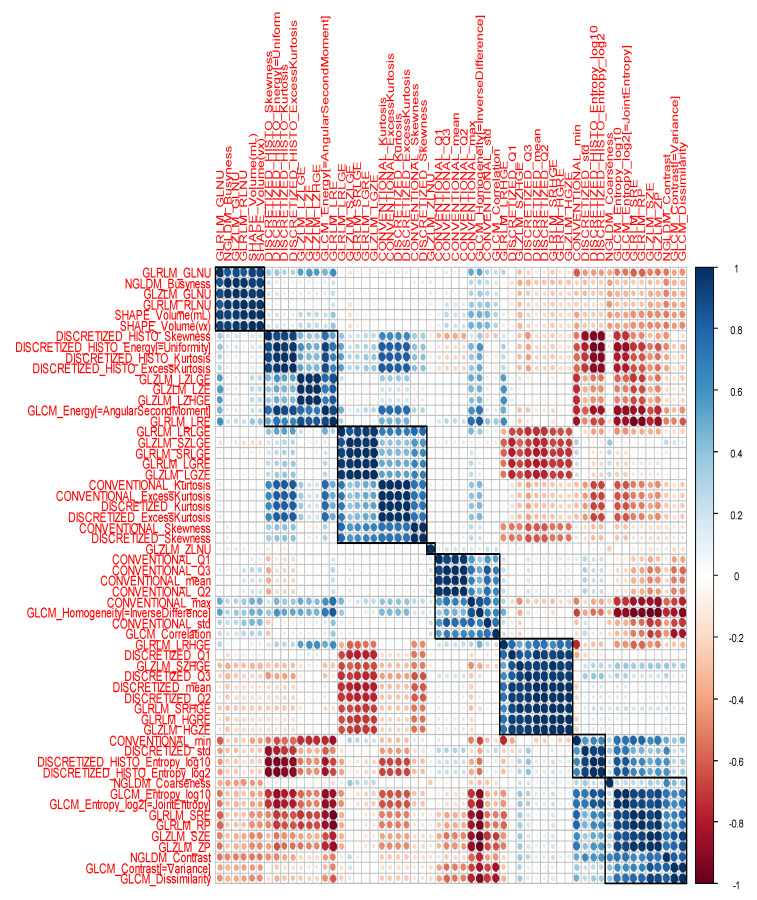
Radiomics heatmap. Heatmap depicting the correlation coefficients matrix of features. Unsupervised clustering analysis was used. Blue was used to represent positive correlations and red to represent negative correlations.

Sixty features were extracted. We removed two features with zero variance, namely, “DISCRETIZED_min” and “DISCRETIZED_max”; therefore, the associations between the 58 selected features and the study endpoint were eventually investigated. A heatmap depicting the Spearman correlation matrix of features is shown in Figure 2, showing high correlations between groups of features.

Two highly correlated features (rho = 0.82) were associated with ER: GLCM_Contrast and NGLDM_Contrast. The most significant was the latter one (*p* = 0.01 vs. 0.04). Larger values of NGLDM_Contrast were significantly associated with ER positivity. (NGLDM_Contrast*100, OR = 1.13, 95% CI [1.03, 1.25], *p*-value = 0.012, Supplementary Figure S1).

Likewise, NGLDM_Contrast was the most significant feature associated with the PR expression, with larger values of NGLDM_Contrast associated with PR positivity (NGLDM_Contrast* 100, OR = 1.11, 95 CI [1.02, 1.21], *p*-value = 0.01, Supplementary Figure S2). Two lowly correlated features (rho = −0.38) were associated with Ki-67 expression: CONVENTIONAL_std and NGLDM_Coarseness. However, by entering both features into a multivariable model, only NGLDM_Coarseness retained a borderline statistical significance (*p* = 0.06, results not shown), with an inverse association with Ki-67 expression (i.e., the lower the radiomic feature’s value, the lower the probability to have Ki-67 higher than 20%). The univariate OR [95% CI] for NGLDM_Coarseness*1000 was 0.57 [0.35, 0.88], *p*-value = 0.02 (Supplementary Figure S3).

Five highly correlated (rho > 0.80) features were significantly associated with HER2+, namely, SHAPE Volume(mL), SHAPE_Volume(vx), GLRLM_RLNU, NGLDM_Busyness and GLZLM_GLNU. Larger values of these features were significantly associated with HER2+ status (see Table 2).

No significant associations of the radiomic features with triple-negative status and G2–G3 grading were found.

## 3. Discussion

The application of radiomics to clinical practice, in particular the correlation with molecular biomarkers, represents one of the most interesting challenges for radiologists and clinicians in recent years [15,16,17,18]. The possibility of providing increasingly personalized and precise diagnostic and therapeutic pathways can be greatly assisted by the intrinsic radiological image information that radiomics is able to provide: radiomics is able to support the clinical decision-making process of the patient’s management. In this study, we aimed to combine the huge potential of radiomic features applied to CESM, which is a relatively new technique with extremely promising results. This, to our knowledge, is one of the few studies that investigated the application of radiomic analysis to CESM images. We showed that certain radiomic features were associated with a specific type of receptor expression.

The possibility of predicting patients with a less unfavorable tumor histotype (i.e., ER- and PR-positive patients or patients with a low expression of Ki-67) or unfavorable tumor histotype (e.g., patients that are HER2+) before a biopsy could be revolutionary in clinical settings.

In breast units, the workflow could be better organized, for example, by offering a quicker biopsy assessment and histologic grading to patients in whom we expect a worse receptor pattern [19]. Nowadays, the determination of molecular features of a neoplasm is done with a biopsy as the gold standard. This ascertainment is invasive, relatively expensive and does not account for the molecular changes in the neoplasm over time [20]. These kinds of shortcomings could be solved by the possibilities of radiomics applied to CESM to predict a certain type of receptor arrangement.

Many studies confirmed the usefulness of CESM in clinical practice, as well as the usefulness of this relatively new diagnostic method [8,21,22,23]. CESM appears to offer similar diagnostic performance to MRI with the advantage of being cheaper, faster and applicable to more patients (e.g., patients with claustrophobia and pacemakers) [24]. CESM’s low-energy images also provide comparable information to conventional mammography [25].

So far, only a few studies with limited numbers of patients have focused on the applications of radiomics applied to CESM. However, the results of these studies are more than encouraging enough to continue applying efforts in this direction. In particular, Fanizzi et al. [26] showed, in a group of 48 breast lesions, from whom a feature set was extracted in the recombined images of CESM, a good performance in the prediction of the malignancy of a lesion with median values of sensitivity and specificity of 87.5% and 91.7%, respectively. La Forgia et al. [27] showed in a group of 68 lesions a good performance regarding discriminating HER2+/HER2− (90.87%), ER+/ER− (83.79%) and Ki-67+/Ki-67− (84.80%). Encouraging results were also obtained by Marino et al. [28] in a group of 103 breast lesions, where radiomic analysis achieved an excellent performance of 87.4% for differentiating invasive from non-invasive cancers and 78.4% for differentiating HR-positive from HR-negative cancers.

To the best of our knowledge, our study had the highest number of malignant breast lesions (205) analyzed with CESM from which radiomic features were extracted to be associated with the molecular features of the tumor. With univariate logistic regression, we found features associated (*p*-value < 0.05) with the expression of estrogen, progesterone, Ki-67 and HER2, thus confirming the possibility of non-invasively assessing the molecular profile of certain neoplastic lesions.

The main limitations of our study were its single-center nature and the lack of external validation of the radiomic correlations obtained. Although we performed a high number of univariate statistical tests, we allowed unadjusted *p*-values to guide the interpretation of our results. Given the exploratory rather than confirmatory nature of the present study, we believe that our approach of describing the tests of significance we performed is appropriate, as advised by Perneger [29].

The manual segmentation of features could be one limitation of the study: manual segmentation is time-consuming and may present some observer bias. Automated image segmentation could be one excellent alternative option. However, so far, in the few published studies on the subject [30], objective standardization of automated algorithms seems to be difficult and often subject to problems of clinical applicability [30]. Further multicenter studies with external validation of the results and with the possibility of automatic feature segmentation are needed to confirm these preliminary findings.

## 4. Materials and Methods

This monocentric study was conducted according to the guidelines of the Declaration of Helsinki and approved by the Ethics Committees of the European Institute of Oncology (protocol numbers IEO S626/311 and IEO 960; EUDRACT number 2019-000326-22; approval dates 30 March 2012 and 7 September 2020). All the patients signed a specific informed consent form.

We selected all the patients with a breast lesion judged to be deserving of cytological/histological assessment (321 patients with 377 breast lesions BI-RADS > 3) [31] after undergoing FFDM or a US performed in our institution. All the patients enrolled in this study underwent CESM prior to a cytohistological assessment. We selected each patient with a positive histological result and who was referred to undergo breast surgery (*n* = 249).

CESM images were evaluated by a radiologist with more than 20 years of experience in consensus with a second, less experienced radiologist (less than 5 years of experience).

Breast lesion CESM enhancement was assessed according to the intensity of the contrastographic uptake into 4 categories (absent, mild, moderated, marked) [3]. Patients with “no enhancement” were excluded from the study due to the impossibility of extracting radiomic features (*n* = 44).

### 4.1. Clinical Information

Patient data were collected, as well as information about the lesions and histological results of surgery. Biopsy results were recorded for the histology, histological grade, receptor status and Ki-67 proliferation index [32]. We collected data regarding the expression of estrogen receptor (ER), progesterone receptor (PR) [33], human epidermal growth factor receptor 2 (HER2) and Ki-67 antigen via immunohistochemical analysis performed in our pathology department. The tumor grade was also determined according to grades G1 (low grade), G2 (intermediate grade) or G3 (high grade).

For our analysis, we considered ER- and PR-positive (>1%) patients with Ki-67 < 20% to be the patients with the best prognosis [34,35,36,37]. We considered those with the worst prognosis to be the HER2-positive patients [38], triple-negative patients [39] and patients with G2 and G3 grading [40]. A surgical specimen was considered the gold standard for histological analysis and the evaluation of the receptor status.

### 4.2. CESM Examinations

All CESM examinations were performed using a full-field digital mammography system derived from a Pristina™ Mammographer (GE Healthcare, Chalfont St. Giles, UK), which was modified to allow for dual-energy exposures, and dedicated software for image acquisition and processing. An automated single-shot intravenous injection of an iodinated contrast agent (Ioexolo) (300 mg/mL, 1.5 mL/kg, Omnipaque^®^, GE Healthcare) with a flow rate of 3 mL/s was administered to the patient before breast compression. Two minutes after the initiation of the contrast medium injection, a set of bilateral craniocaudal (CC) and mediolateral oblique (MLO) views was acquired, starting with the breast without the suspicious lesion. Images of both views and both breasts were completed within 5 min. Two exposures were acquired, one with low energy (26–32 kVp) and one with high energy (45–49 kVp). The low- and high-energy images were then recombined to highlight the uptake of the contrast agent.

### 4.3. Image Analysis

Two radiologists in consensus evaluated the CESM images and classified the grade of enhancement of the lesions (absent, mild, moderated, marked).

The readers were blinded to other imaging findings and clinical information (side of breast cancer, symptoms, medical history). As the CESM examination provides a pair of images for each view and each breast for image interpretation, i.e., the low energy image and the recombined images displaying contrast enhancement, both were used for diagnosis.

Each detected lesion was specified according to the BI-RADS classification [41], localization and lesion size (maximum diameter) on each imaging investigation.

The maximum dimension of the CESM-detected lesions was measured on the recombined images based on the contrast uptake, taking anatomical findings on the low-energy image into consideration; the results were reported in the case report form.

### 4.4. Radiomic Analysis

For each patient, lesion laterality, lesion size (maximum diameter in either the CC or MLO view), breast density and background parenchymal enhancement were recorded.

In the case of multifocal disease, each lesion was analyzed as a stand-alone lesion.

For the radiomic analysis, a fellowship-trained radiologist with over 4 years of experience in breast imaging manually delineated the borders of the enhancement of each lesion, namely, the region of interest (ROI), to include the entire enhancing lesion and to exclude background enhancement.

**Figure 3:**
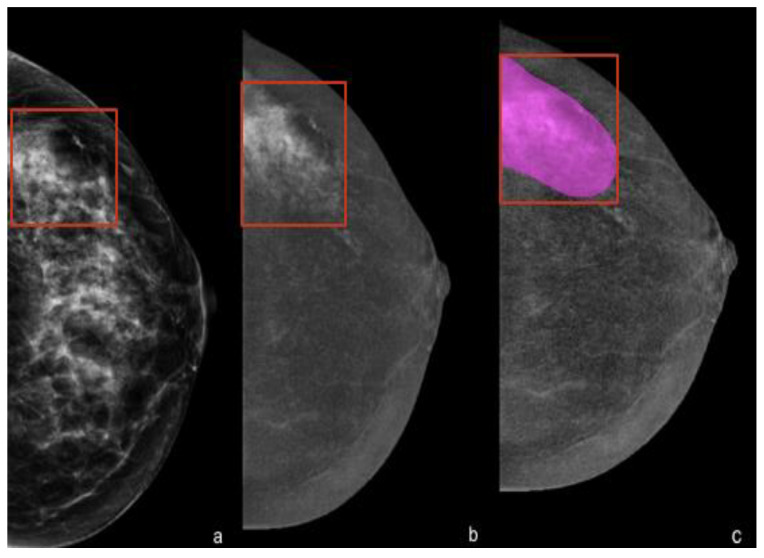
Delineation of contours showing marked enhancement. (**a**) Left cranio-caudal full-field digital mammography showing an extended cluster of microcalcifications in the outer quadrants that were highly suspicious for malignancy (in the red box). (**b**) Cranio-caudal contrast-enhanced mammography showing minimal background parenchymal enhancement and a marked heterogeneous non-mass enhancement at the site of the microcalcifications. (**c**) Manual segmentation of the contrast-enhanced area for the radiomic analysis. The final histology revealed a ductal carcinoma in situ (DCIS), ER- and PR-positive, HER2-negative, G3, Ki-67 24%.

**Figure 4:**
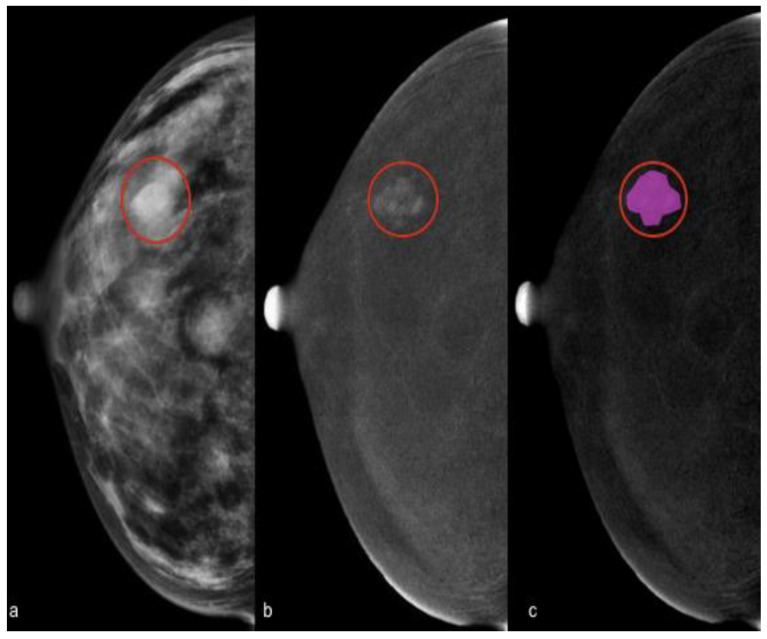
Delineation of contours showing moderate enhancement. (**a**) Right cranio-caudal full-field digital mammography showing a fibroglandular breast (ACR: D) with multiple focal opacities. (**b**) Cranio-caudal contrast-enhanced mammography showing minimal background parenchymal enhancement and a moderated mass enhancement in the outer quadrant in correspondence with one of the opacities seen in the full-field digital mammography (in the red circle). (**c**) Manual segmentation of the contrast-enhanced area for the radiomic analysis. The final histology of the enhanced lesion revealed a ductal invasive carcinoma, ER- and PR-positive, HER2-negative, G2, Ki-67 25%.

Contours were delineated on either the cranio-caudal or medio-lateral-oblique view, but for the radiomic analysis, we decided to consider only one view by default (cranio-caudal). See Figure 3 and Figure 4.

All Digital Imaging and Communications in Medicine (DICOM) images were transferred to a database and loaded onto the open-source image processing tool LIFEx v6.32. Features were extracted with a fixed bin number (64 bins) and spatial resampling to account for different fields of view (FOVs).

### 4.5. Statistical Analysis

Characteristics of patients were summarized with median and interquartile range (IQR) for quantitative variables and with frequency and percentage for categorical variables.

All features with zero variance were excluded from the analysis. The Spearman correlation matrix of the features was calculated and graphically represented with a heatmap using the *ComplexHeatmap* package in R.

The association of each feature with any of the evaluated endpoints (ER, PR, Ki-67, HER2+, triple-negative, G2–G3 expressions) was assessed through logistic regression models and quantified in terms of odds ratios (ORs). If more than one feature was significantly associated with one of the endpoints, then the Spearman correlation between the features was calculated. If the correlation was higher than 0.8, then only the feature most associated with the outcome (defined as the feature with the smallest *p*-value in the logistic regression) was considered predictive. Where appropriate, features were rescaled to obtain interpretable ORs.

All analyses were carried out using R, version 4.1.2.

## 5. Conclusions

According to our results, the radiomic features of CESM images could be helpful for predicting particular molecular subtypes before a biopsy: NGLDM_Contrast was positively associated with both ER and PR expression (*p*-values = 0.01); NGLDM_Coarseness was negatively associated with Ki-67 expression (*p*-value = 0.02); and five highly correlated (rho > 0.80) features were significantly associated with HER2+, namely, SHAPE Volume(mL), SHAPE_Volume(vx), GLRLM_RLNU, NGLDM_Busyness and GLZLM_GLNU.

Our study included a considerable number of breast lesions and is one of the few studies that investigated the application of radiomic analysis to CESM images. We showed the first non-invasive and quick step toward the prediction of key receptor biomarkers of a neoplasm by combining radiomic feature extraction with a promising new diagnostic method, namely, CESM. Such a possibility could allow patients with a worse prognosis to be identified much earlier and referred for timely diagnostic and therapeutic management.

## Figures and Tables

**Table 1:** Descriptive variables of the patients and lesions.

Type of Lesion *n* (%)	
Architectural distortion	3 (1.5)
Enhancement MRI	4 (2.0)
Mass	173 (84)
Mass with microcalcifications	4 (2.0)
Microcalcifications	17 (8.3)
Missing information	4 (2.0)
Age median (IQR)	50 (45–58)
BI-RADS (*n*%)	
4a	8 (4.0)
4b	49 (24)
4c	88 (44)
5	57 (28)
Missing	3
Density (ACR) (*n*%)	
A	2 (1.0)
B	44 (21)
C	140 (68)
D	19 (9.3)
Background (*n*%)	
Marked	10 (4.9)
Mild	49 (24)
Minimal	127 (62)
Moderated	19 (9.3)
Enhancement intensity (*n*%)	
Marked	92 (45)
Mild	31 (15)
Moderated	82 (40)
Median size of the enhanced lesion (IQR)	17 (11–30)
ER+	178 (89%)
Missing	5
PR	170 (85%)
Missing	5
Ki-67	106 (53%)
Missing	5
HER2+	39 (20%)
Missing	5
Grading+	168 (84%)
Missing	5
Triple-negative	30 (15%)
Missing	5

IQR—interquartile range.

**Table 2:** Univariate logistic regression between the frequencies of the listed features and HER2.

	OR ^1^	95% CI ^1^	*p*-Value
SHAPE Volume(mL)/100	1.66	1.06, 2.61	0.025
SHAPE_Volume(vx)/10,000	1.05	1.01, 1.10	0.025
GLRLM_RLNU/1000	1.01	1.00, 1.01	0.016
NGLDM_Busyness	1.16	1.03, 1.32	0.020
GLZLM_GLNU/1000	1.25	1.03, 1.52	0.023

^1^ OR—odds ratio, CI—confidence interval.

## Data Availability

The data presented in this study are available upon request from the corresponding author. The data are not publicly available due to privacy concerns, in accordance with the GDPR.

## Supplementary Materials

The supporting information can be downloaded from https://www.mdpi.com/article/10.3390/ijms232315322/s1.
