# Supplementary material for: Radiomic Features Applied to Contrast Enhancement Spectral Mammography: Possibility to Predict Breast Cancer Molecular Subtypes in a Non-Invasive Manner
Source: Int J Mol Sci. 2022 Dec 5;23(23):15322. doi: 10.3390/ijms232315322 (PMC9740943; doi:10.3390/ijms232315322)
Supplement: Supplementary file 1 [file ijms-23-15322-s001.zip › Supplementary.pdf]

Supplementary Figure S1

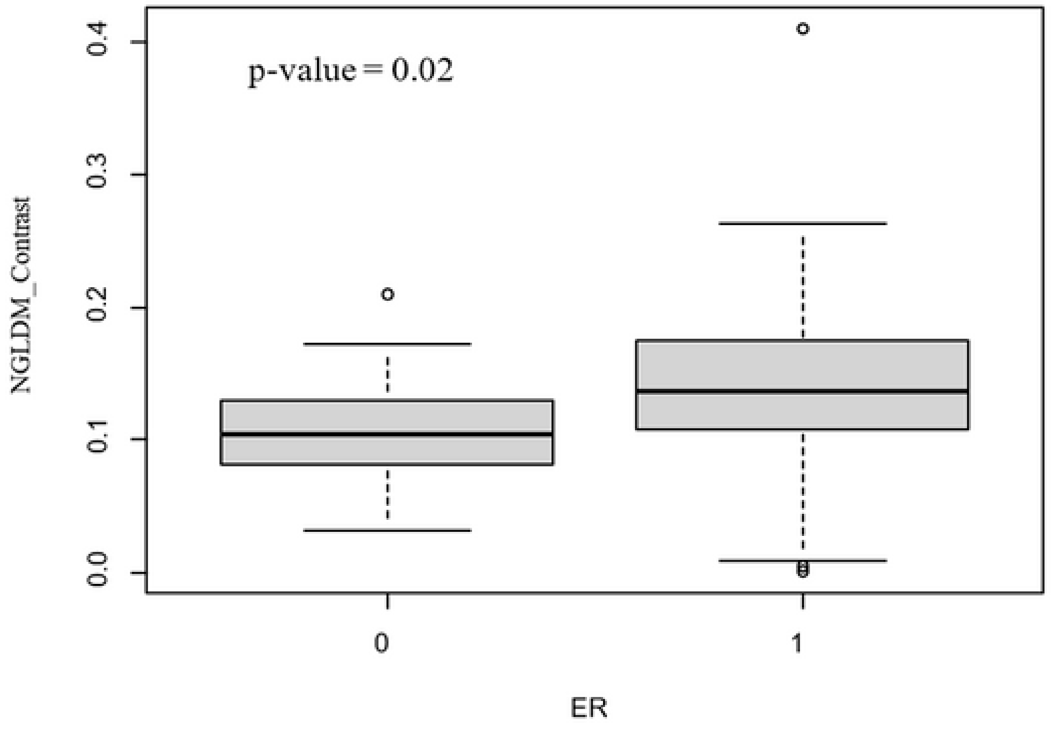

Supplementary Figure S2

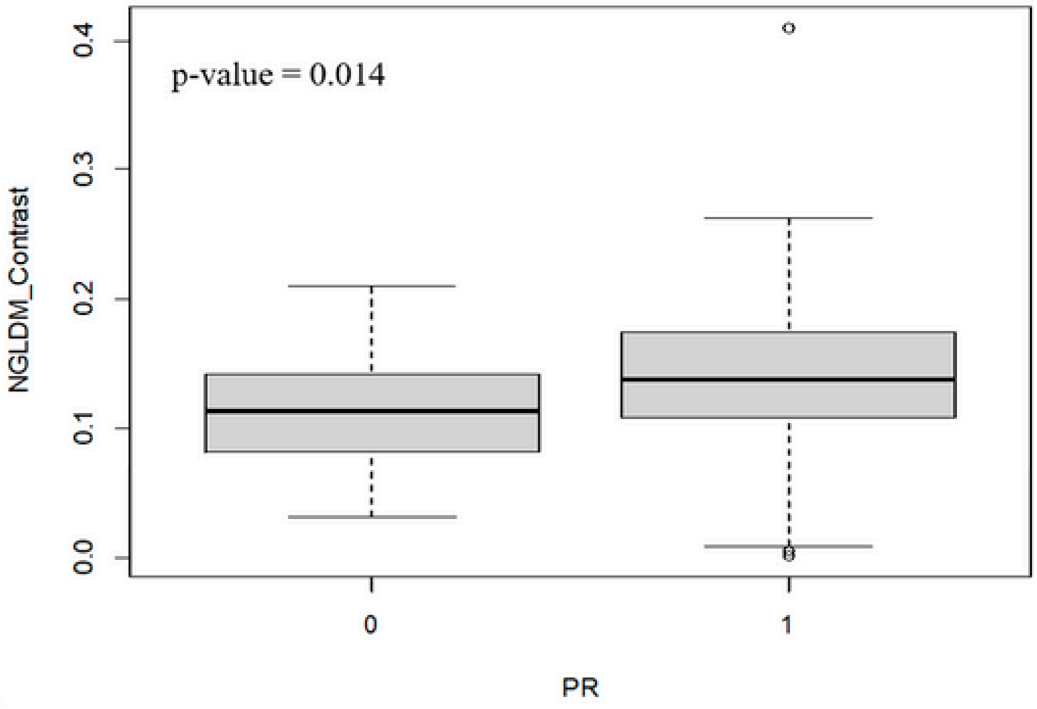

Supplementary Figure S3

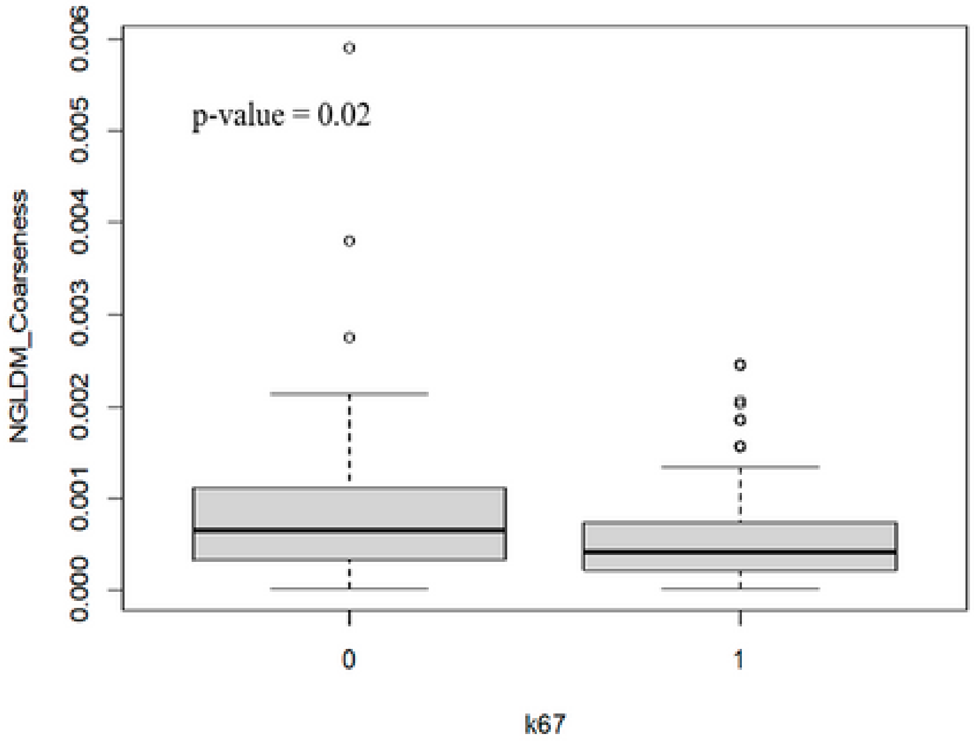

Supplementary Table S1: Histological results of surgery.

**Biopsy results (n%)**	
Cribriform carcinoma	4 (2.0)
Invasive mixed (lobular and ductal) carcinoma	9 (4.4)
Malignant phylloid	1 (0.5)
Micropapillary carcinoma	1 (0.5)
Acinic cell carcinoma	2 (1.0)
High- grade ductal carcinoma in situ	6 (2.9)
Low- grade ductal carcinoma in situ	4 (2.0)
Intermediate- grade ductal carcinoma in situ	11 (5.3)
Invasive ductal carcinoma	139 (67.8)
Invasive lobular carcinoma	27 (13.1)
Mucinous carcinoma	1 (0.5)
